# Effects of a preconception lifestyle intervention in obese infertile women on diet and physical activity; A secondary analysis of a randomized controlled trial

**DOI:** 10.1371/journal.pone.0206888

**Published:** 2018-11-07

**Authors:** Tessa M. van Elten, Matty D. A. Karsten, Anouk Geelen, Anne M. van Oers, Mireille N. M. van Poppel, Henk Groen, Reinoud J. B. J. Gemke, Ben Willem Mol, Meike A. Q. Mutsaerts, Tessa J. Roseboom, Annemieke Hoek, on behalf of the LIFEstyle study group

**Affiliations:** 1 Amsterdam UMC, Vrije Universiteit Amsterdam, VU University medical center, Department of Public and Occupational Health, de Boelelaan, Amsterdam, The Netherlands; 2 Amsterdam UMC, University of Amsterdam, Academic Medical Center, Department of Clinical Epidemiology, Biostatistics and Bioinformatics, Meibergdreef, Amsterdam, The Netherlands; 3 Amsterdam UMC, University of Amsterdam, Academic Medical Center, Department of Obstetrics and Gynecology, Meibergdreef, Amsterdam, The Netherlands; 4 Amsterdam Public Health Research Institute, Amsterdam, The Netherlands; 5 Amsterdam Reproduction and Development, Amsterdam, The Netherlands; 6 University of Groningen, University Medical Center Groningen, Department of Obstetrics and Gynecology, Groningen, the Netherlands; 7 Wageningen University & Research, Division of Human Nutrition, Wageningen, The Netherlands; 8 University of Graz, Institute of Sport Science, Graz, Austria; 9 University of Groningen, University Medical Center Groningen, Department of Epidemiology, Groningen, the Netherlands; 10 Amsterdam UMC, Vrije Universiteit Amsterdam, Emma Childrens Hospital, Department of Pediatrics, Amsterdam, The Netherlands; 11 Monash University, Department of Obstetrics and Gynecology, Melbourne, Australia; TNO, NETHERLANDS

## Abstract

**Background:**

Lifestyle changes are notoriously difficult. Since women who intend to become pregnant are more susceptible to lifestyle advice, interventions during this time window might be more effective than interventions during any other period in life. We here report the effects of the first large preconception lifestyle intervention RCT on diet and physical activity in obese infertile women.

**Methods:**

In total, 577 women were randomized between a six-month lifestyle intervention program (intervention group; N = 290) or prompt infertility treatment (control group; N = 287). Self-reported dietary behaviors and physical activity were assessed at baseline, three, six and twelve months after randomization. Mixed models were used to analyze differences between groups.

**Results:**

Compared to the control group, the intervention group reduced their intake of sugary drinks at three months (-0.5 glasses/day [95% C.I. = -0.9;-0.2]), of savory snacks at three (-2.4 handful/week [-3.4;-1.4]) and at six months (-1.4 handful/week [-2.6;-0.2]), and of sweet snacks at three (-2.2 portion/week [-3.3;-1.0]) and twelve months after randomization (-1.9 portion/week [-3.5;-0.4]). Also, the intervention group was more moderate to vigorous physically active at three months after randomization compared to the control group (169.0 minutes/week [6.0; 332.1]).

**Conclusion:**

Our study showed that obese infertile women who followed a six-month preconception lifestyle intervention program decreased their intake of high caloric snacks and beverages, and increased their physical activity. These changes in lifestyle may not only improve women’s health but their offspring’s health too.

## Introduction

The increasing prevalence of obesity is a major public health problem in women of reproductive age [1]. Besides the association of obesity with increased prevalence of non-communicable diseases [2], it also adversely affects women’s reproductive health [3,4], as well as offspring’s health [5].

A healthy lifestyle is recommended as the first step to control obesity [6]. However, we do know that structurally improving lifestyle is notoriously difficult. Women who intend to become pregnant are known to be more susceptible to lifestyle advice, for example to quit smoking and stop drinking alcohol [7,8]. Therefore, lifestyle interventions prior to conception might be more effective in changing diet and physical activity than interventions during any other period in life.

Up until now, studies mainly focused on intervening during the period of pregnancy [9–14], but currently attention shifts to intervention strategies targeting obese women before pregnancy to improve reproductive, maternal and child health [15–17]. However, no experimental studies assessing the effect of preconception lifestyle interventions in humans have been done yet.

The LIFEstyle study was the first randomized controlled trial (RCT) designed to examine the efficacy of a preconception lifestyle intervention in a large group of obese infertile women on reproductive, gestational and delivery outcomes [18]. The lifestyle intervention resulted in significantly more weight loss [19] and improved cardiometabolic health [20], but it is unclear how the intervention changed lifestyle.

Therefore, we here report the effects of the LIFEstyle preconception intervention program on diet and physical activity in obese infertile women throughout the intervention program and thereafter.

## Materials and methods

The LIFEstyle study was a multicenter RCT in obese infertile women (Dutch trial register; NTR 1530; http://www.trialregister.nl/trialreg/admin/rctview.asp?TC=1530). Participants were included in the study between June 9, 2009 and June 22, 2012 and followed for two years. Design and primary results of the LIFEstyle study have been described previously [18,19]. In brief, the original study population consisted of 577 infertile women between 18 and 39 years old, with a BMI of ≥29 kg/m^2^. Women were eligible for recruitment when presenting with infertility in a general or academic hospital. Infertility was defined as failure to conceive within 12 months of unprotected intercourse in case of an ovulatory cycle, or in case of chronic anovulation according to WHO class I or II. Couples were excluded if suffering from azoospermia or using donor semen, women with endometriosis AFS class III or IV, chronic anovulation WHO class III (premature ovarian failure) or endocrinopathies (such as Cushing syndrome, adrenal hyperplasia and diabetes type I). Women with untreated pre-existent hypertension, preeclampsia, eclampsia or HELLP syndrome in a previous pregnancy were also not eligible.

This study was conducted according to the guidelines laid down in the Declaration of Helsinki. All procedures were approved by the Medical Ethics Committee of the University Medical Center Groningen, the Netherlands (METc 2008/284) and the review board of each participating center. Written informed consent was obtained from all participants.

### Intervention

Participants were randomized by a web-based randomization program at a central location, stratified according to trial center and ovulatory status. Blinding was not possible due to the nature of the intervention. Participants randomized into the intervention arm participated in a six-month structured lifestyle program, aiming at a weight loss of 5–10% of the original body weight. After completion of the intervention program, if the target weight reduction of 5–10% was met, or if BMI decreased below 29 kg/m^2^, infertility treatment was started in accordance with the Dutch infertility guidelines [21]. When becoming pregnant participants discontinued the intervention, but they could re-enter the intervention in case of a miscarriage. The control group promptly started infertility treatment based on the Dutch infertility guidelines. They did not receive any lifestyle advice with the exception of the patient information leaflet containing general information on the adverse effects of overweight and obesity on women’s reproductive health, pregnancy, and pregnancy outcomes.

The lifestyle program combined counselling on diet and physical activity with an individualized behavioral modification plan [22–24]. Intervention nurses, with a background in infertility care, were trained to guide and support the participants during six face-to-face and four telephone consultations [18]. Participants were advised to consume a healthy diet according to the Dutch dietary guidelines of 2006 [25] with a caloric reduction of approximately 600kcal compared to their usual caloric intake, but not below 1200kcal/day. To create awareness of total food intake, participants could receive feedback on food and caloric intake on a daily basis using a web-based food diary of the Netherlands Nutrition Center [26]. Participants brought a copy of these results to the consultations to discuss their dietary intake. In addition, participants were advised to be physically active 2–3 times a week for at least 30 minutes at moderate intensity (60–85% of maximum heart rate frequency), and to increase physical activity in daily life by taking 10.000 steps per day monitored with a pedometer. A diary was kept on these physical activities to establish self-monitoring, which was also used during the consultations to discuss physical activity levels.

### Diet

Participants in both the intervention and the control group were asked to complete a food frequency questionnaire (FFQ) four times. Once at the start of the intervention, and at three, six and twelve months after randomization. The self-administered FFQ asked about foods and food groups the intervention focused on. It consisted of two parts: the first part includes the standardized questionnaire on food consumption used for the Public Health Monitor in the Netherlands [27]. This first part has been supplemented with a second part, consisting of additional frequency and portion size questions about snack intake and the usage of sugar containing and alcoholic beverages. Frequency of consumption was asked per week or per month. Portion size for all foods and food groups had been asked per standard household measure (e.g. glass or handful). We focused on the intake of vegetables (raw as well as cooked; grams/day), fruits (grams/day), sugary drinks (fruit juice and soda; glasses/day), alcoholic beverages (glasses/day) and the intake of savory snacks (crisps, pretzels, nuts and peanuts; handful/week) and sweet snacks (biscuits, pieces of chocolate, candies or liquorices; portion/week). One portion of sweet snacks included 2 biscuits, or 2 pieces of chocolate, or 5 candies, or 5 pieces of liquorice. Portion sizes and food groups as presented were pre-specified in the questions of the FFQ.

### Physical activity

Participants completed the Short QUestionnaire to ASsess Health-enhancing physical activity (SQUASH) four times. Once at the start of the intervention, and at three, six and twelve months after randomization. The SQUASH is a validated questionnaire to rank subjects according to their level of physical activity [28]. Data were collected about commuting activities, leisure time activities, household activities, and activities at work and school, using three main questions: days per week, average time per day/week (hours and/or minutes), and intensity (low, moderate, high). We focused on the outcomes moderate to vigorous leisure time physical activity (minutes/week), moderate to vigorous commuting activities (walking or cycling from/to work or school; minutes/week) and moderate to vigorous total physical activity (MVPA; minutes/week).

### Statistical methods

Differences and 95% confidence intervals (95% C.I.) in dietary intake as well as in physical activity between both groups at three, six and twelve months after randomization were analyzed by mixed model analysis, using a random intercept. This method was chosen to account for decreasing response to questionnaires over time. All associations were adjusted for baseline values, using time and an interaction term between time and randomization group in the model. In addition, results are expressed as marginal means per time point, incorporating the dependency of observations within subjects and corrections for baseline. We checked if our data was normally distributed after adjusting for baseline values. To identify potential confounders, we adjusted for pregnancy, education level and smoking, one at the time, because of small, statistically non-significant differences between intervention and control group at baseline. If the effect estimate in the majority of the models changed >10%, we included the variable in the final model. To account for differences in the number of pregnant women in the intervention and control group, we tested for effect modification by adding pregnancy to the model and an interaction term with randomization group. Alcoholic beverages and commuting activities both had a median of zero in combination with a very narrow distribution, therefore we only showed medians and inter quartile rangers (IQR) for these variables (S2 and S3 Tables).

We additionally used univariate regression models to explore if weight change between baseline and six months after randomization (clinically measured weight in kg at 6 months minus clinically measured weight in kg at baseline) was related to changes in diet and physical activity between baseline and six months after randomization (physical activity/diet at 6 months minus physical activity/diet at baseline). Only total MVPA and diet variables that were statistically significant in our mixed model analyses were included. We performed these explorative analyses irrespective of randomization group, using complete cases while pregnant women were excluded.

All questions of the FFQ contained open answer categories for the largest portion size (e.g. more than 5 glasses of soda), with the exception of vegetable intake. As we did not know the exact portion size consumed when this answer was given, we arbitrarily chose to recode the portion size for these categories into X+1 (e.g. 6 glasses of soda). We performed a sensitivity analysis with X+1+30% (e.g. 8 glasses of soda) and found that the associations were robust (S1 Table).

Statistical analyses were performed using the software Statistical Package for the Social Sciences (SPSS) version 22 for Windows (SPSS, Chicago, IL, USA). P-values <0.05 were considered statistically significant.

## Results

**Fig 1:**
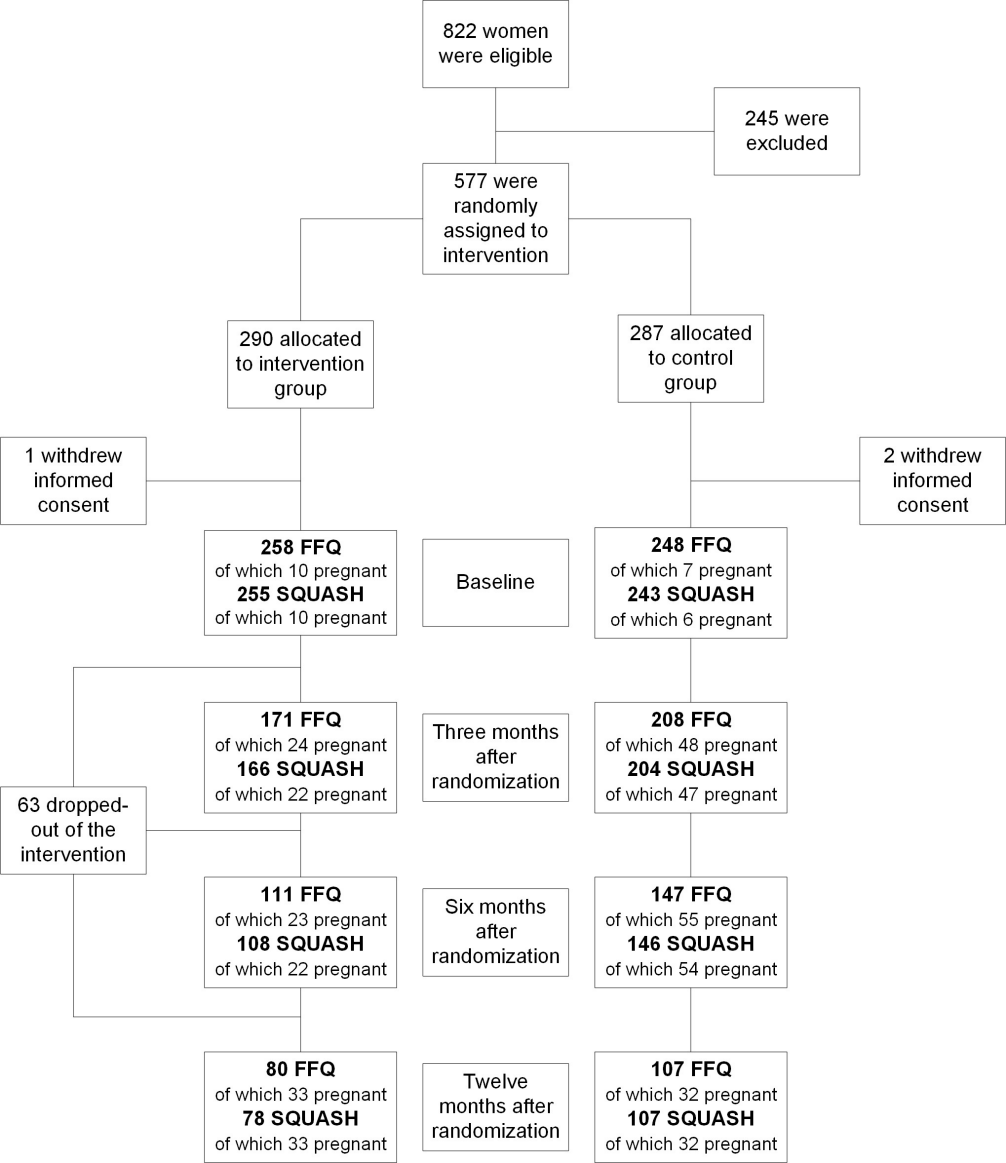
Flow diagram LIFEstyle study for diet and physical activity data. FFQ = Food Frequency Questionnaire; SQUASH = Short QUestionnaire to ASsess Health-enhancing physical activity; mo. = months.

Table 1 shows the characteristics of the study participants who completed the FFQ and/or SQUASH at baseline (N = 510). Characteristics were similar for the intervention group and the control group. There were no differences compared to the LIFEstyle study participants as a whole (N = 574). Response decreased over time for both questionnaires (Fig 1). S2 and S3 Tables show the dietary intakes and physical activity at baseline, three, six and twelve months after randomization. After correction for baseline values, residuals were normally distributed. For diet and physical activity we found no significant interaction effect between pregnancy and randomization group. Therefore, our model does not include an interaction term between pregnancy and randomization group. Results were adjusted for pregnancy, education level and smoking based on their impact on the effect estimates.

**Table 1:** Characteristics of participants who completed the FFQ and/or SQUASH at baseline.

	Intervention group (N = 261)	Control group (N = 249)	P-value
Age (mean; SD)	29.8 (4.5)	29.8 (4.5)	0.88
Caucasian (%; N)	89.3 (233)	89.2 (222)	0.97
Education (%; N)			
Primary school (4–12 years)	6.0 (15)	2.9 (7)	0.26
Secondary education	24.0 (60)	23.4 (56)	
Intermediate Vocational Education	49.2 (123)	47.7 (114)	
Higher Vocational Education and University	20.8 (52)	25.9 (62)	
Smoking (yes; %; N)	26.1 (67)	21.4 (53)	0.22
Weight (kg; mean; SD)	103.7 (13.7)	103.4 (12.3)	0.80
Body Mass Index (kg/m^2^; mean; SD)	36.0 (3.4)	36.1 (3.4)	0.85
Anovulation (yes; %; N)	45.0 (117)	48.4 (120)	0.44
PCOS (%; N)	76.1 (89/117)	74.2 (89/120)	0.70
Nulliparous (%; N)	70.1 (183)	67.1 (167)	0.73

Baseline characteristics are presented as means and standard deviations (SD) for continuous variables, and as percentages (%) and total number of participants (N) for categorical data. To compare groups, an independent Student’s t-test was used for continuous variables, and a Chi-square test for categorical data; kg/m2 = kilograms per square meter; PCOS = Polycystic ovarian syndrome.

### Diet

Table 2 shows the overall differences in lifestyle between the intervention and control group, which represents the effect of randomization group on the diet and physical activity outcomes irrespective of the effect of time, and the differences in lifestyle per time point after randomization. There were overall group effects for the intake of sugary drinks (-0.4 glasses/day [95% C.I. = -0.6; -0.1]; Table 2), savory snacks (-1.8 handful/week [-2.6; -0.9]), and sweet snacks (-1.8 portion/week [-2.8; -0.9]). The intervention group had a lower intake of sugary drinks at three months after randomization compared to the control group (-0.5 glasses/day [-0.9; -0.2]). They also had a lower intake of savory snacks at three months (-2.4 handful/week [-3.4; -1.4]) and at six months after randomization (-1.4 handful/week [-2.6; -0.2]), and a lower intake of sweet snacks at three months (-2.2 portion/week [-3.3; -1.0]) and twelve months after randomization (-1.9 portion/week [-3.5; -0.4]) compared to the control group.

**Table 2:** Differences in diet and physical activity in the intervention group compared to the control group.

	Overall (95% C.I.)^a^	Time point after randomization	Difference (95% C.I.)		P-value
**Vegetable intake (gram/day)**					
Corrected for baseline	6.3 (-4.1; 16.6)	Three months	5.2 (-6.9; 17.4)		0.40
		Six months	13.2 (-1.0; 27.4)		0.07
		Twelve months	-3.3 (-19.2; 12.6)		0.69
Corrected for baseline, education, pregnancy and smoking	4.0 (-6.8; 14.8)	Three months	3.1 (-9.5; 15.7)		0.63
		Six months	10.7 (-4.1; 25.6)		0.16
		Twelve months	-4.9 (-21.6; 11.7)		0.56
**Fruit intake (gram/day)**					
Corrected for baseline	-0.5 (-11.8; 10.8)	Three months	7.2 (-6.8; 21.2)		0.32
		Six months	-12.3 (-28.9; 4.2)		0.14
		Twelve months	-0.7 (-19.6; 18.2)		0.94
Corrected for baseline, education, pregnancy and smoking	0.7 (-10.8; 12.3)	Three months	8.9 (-5.3; 23.1)		0.22
		Six months	-8.7 (-25.5; 8.2)		0.31
		Twelve months	-5.3 (-24.6; 14.0)		0.59
**Sugary drinks (glasses/day)**					
Corrected for baseline	-0.4 (-0.7; -0.1)^c^	Three months	-0.5 (-0.9; -0.2)		0.001
		Six months	-0.5 (-0.8; -0.1)		0.03
		Twelve months	0.02 (-0.4; 0.5)		0.93
Corrected for baseline, education, pregnancy and smoking	-0.4 (-0.7; -0.1)^c^	Three months	-0.6 (-0.9; -0.2)		0.001
		Six months	-0.4 (-0.8; 0.02)		0.07
		Twelve months	-0.04 (-0.5; 0.4)		0.86
**Savory snacks (handful/week)**					
Corrected for baseline	-1.8 (-2.7; -1.0)^d^	Three months	-2.4 (-3.4; -1.4)		<0.001
		Six months	-1.5 (-2.7; -0.3)		0.01
		Twelve months	-0.8 (-2.1; 0.5)		0.25
Corrected for baseline, education, pregnancy and smoking	-1.7 (-2.6; -0.9)^d^	Three months	-2.5 (-3.5; -1.5)		<0.001
		Six months	-1.4 (-2.6; -0.2)		0.03
		Twelve months	-0.4 (-1.8; 0.9)		0.52
**Sweet snacks (portion/week)**^**b**^					
Corrected for baseline	-1.9 (-2.8; -1.0)^d^	Three months	-2.3 (-3.4; -1.1)		<0.001
		Six months	-1.4 (-2.8; -0.1)		0.04
		Twelve months	-1.8 (-3.3; -0.2)		0.03
Corrected for baseline, education, pregnancy and smoking	-1.8 (-2.8; -0.9)^d^	Three months	-2.2 (-3.3; -1.0)		<0.001
		Six months	-1.2 (-2.6; 0.2)		0.08
		Twelve months	-1.8 (-3.4; -0.2)		0.03
**Total moderate to vigorous physical activity (min/week)**					
Corrected for baseline	132.0 (5.5; 258.6)^c^	Three months	172.7 (14.9; 330.5)		0.03
		Six months	91.8 (-94.9; 278.5)		0.34
		Twelve months	57.5 (-155.5; 270.6)		0.60
Corrected for baseline, education, pregnancy and smoking	133.6 (3.0; 264.3)^c^	Three months	169.0 (6.0; 332.1)		0.04
		Six months	93.2 (-102.0; 288.4)		0.35
		Twelve months	81.0 (-141.8; 303.8)		0.48
**Leisure time moderate to vigorous physical activity (min/week)**					
Corrected for baseline	82.4 (-0.2; 165.0)	Three months	107.0 (-2.3; 216.2)		0.06
		Six months	74.1 (-56.3; 204.5)		0.27
		Twelve months	19.0 (-130.9; 168.9)		0.80
Corrected for baseline, education, pregnancy and smoking	63.8 (-21.5; 149.1)	Three months	88.6 (-24.0; 201.3)		0.12
		Six months	49.9 (-86.2; 186.1)		0.47
		Twelve months	12.8 (-143.8; 169.4)		0.87

Differences and 95% confidence intervals (95% CI) were analyzed by mixed model analysis, including all women with at least one value (range N = 511 for sugary drinks; N = 535 for fruit intake), using a random intercept. Time and an interaction term between time and randomization group was used in all models. As all women had different dietary intakes and physical activity levels at baseline, we corrected by default for baseline values. The fully corrected model included correction for the confounders education, pregnancy and smoking; C.I. = confidence interval; min/week = minutes per week.

^a^ The overall effect represents the effect of randomization group on the diet and physical activity outcomes irrespective of the effect of time. The linear mixed model included randomization group, baseline dietary intake/physical activity, and in case of the fully corrected model, education level and pregnancy as independent fixed effect variables. Time was not added to this model.

^b^ One portion of sweet snacks included 2 biscuits, or 2 pieces of chocolate, or 5 candies, or 5 pieces of liquorice.

^c^ P-value <0.05

^d^ P-value <0.001

**Fig 2:**
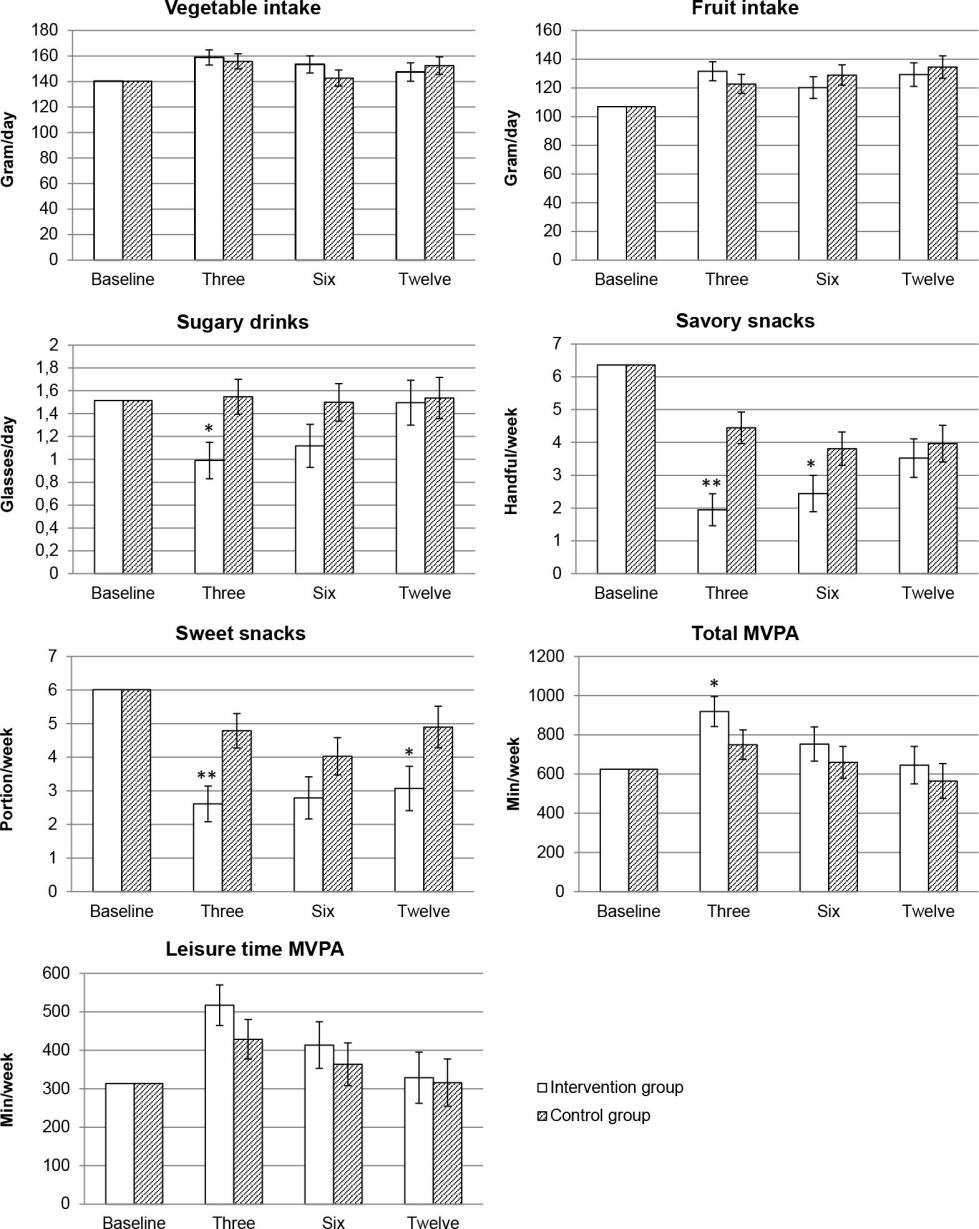
Estimated marginal means for diet and physical activity corrected for baseline, education level, pregnancy and smoking. Marginal means were estimated by mixed model analysis and time was added as a categorical variable into the model. Time points are at baseline, three months, six months and twelve months after randomization in both groups; MVPA = moderate to vigorous physical activity; min/week = minutes per week; * P<0.05, ** P<0.001.

Fig 2 shows the estimated marginal means for dietary intake and physical activity in the intervention and control group over the different time points. We tested if the effects of the intervention on the dietary intake and physical activity outcomes differed over time by adding an interaction term between time and randomization group into our model. Interaction effects between time and randomization group showed no significant results, with exception of savory snacks (p = 0.01). This is due to the large decrease in savory snack intake in the intervention group compared to the control group at three months after randomization (Fig 2).

Explorative univariate regression analyses showed that weight loss during the first six months is related to decreased savory snack intake during the first six months after randomization (mean predicted value = -2.60 handful/week; P = 0.01; total N = 127). No other statistically significant associations between change in body weight and change in lifestyle behaviors were seen.

### Physical activity

There was an overall group effect for total MVPA (133.6 minutes/week [3.0; 264.3]), but not for leisure time MVPA (Table 2). For total MVPA the difference between the intervention group and the control group was statically significant at three months after randomization (169.0 minutes/week [6.0; 332.1]). Thereafter, differences between the intervention group and the control group decreased, although the intervention group was more physically active compared to the control group at all points in time. A similar pattern was seen in leisure time MVPA, but there were no statistically significant differences between the intervention and control group. Interaction effects between time and randomization group showed no significant results.

## Discussion

The six-month structured preconception lifestyle intervention decreased the intake of sugary drinks, sweet and savory snacks in obese infertile women while it did not affect intake of fruit and vegetables. This decreased intake of sweet snacks persisted up to six months after the intervention program ended. Women in the intervention group were more physically active than the women in the control group. Although our study showed modest effects on diet and physical activity outcomes, cardiometabolic health of women improved by halving the odds of metabolic syndrome [20].

The LIFEstyle study was the first large RCT studying the effects of a lifestyle intervention program that starts prior to conception in obese women. We observed the largest intervention effects on diet and physical activity at three months after randomization. A reason for this finding could be that during these first three months, participants had more close contact with the intervention nurse compared to the last three months of the intervention period (6 visits of which 4 face-to-face vs. 4 visits of which 2 face-to-face respectively). Women who attended a greater number of scheduled visits with the intervention nurse more often successfully lost ≥5% of their original bodyweight [29]. Therefore, it seems that the higher intensity of guidance in the first three months of the intervention program encouraged healthy changes in diet and physical activity. In our explorative regression analyses, we found that weight loss during the first six months after randomization was associated with a decreased savory snack intake during these first six months, suggesting that the intervention was mainly effective in achieving weight loss through reduced snacking. Since the focus of our intervention program was weight loss, and therefore to eat less calories and increase physical activity, we hypothesize this could explain the decreased intake of snacks and sugary drinks and the lack of intervention effect on the intake of vegetables and fruit. The lack of maintenance in lifestyle changes at twelve months after randomization (six months after the intervention ended) are in line with studies examining long-term weight loss by diet, exercise or combined diet and exercise programs [30,31].

Studies on lifestyle changes, including diet and physical activity, in women of reproductive age mostly focused on the pregnancy period to improve maternal health and to improve pregnancy outcomes [9–14,32]. Reviews and meta-analyses on these studies show positive effects of lifestyle interventions on restricting gestational weight gain [9,11–13] and trends towards [11], or slightly reduced prevalence of gestational diabetes [14]. Recent RCT’s of lifestyle interventions in pregnant women, the RADIEL, UPBEAT, DALI and LIMIT trial, showed that interventions during pregnancy were effective in altering diet and physical activity [33–38].

Our population consisted of infertile women visiting the gynecologist to start infertility treatment. Therefore, motivations and barriers for changing physical activity and diet might be different than in pregnant women. An important motivation for lifestyle changes during pregnancy is having the responsibility for the health of the unborn child besides personal health [39]. As the women included in the LIFEstyle study were not pregnant yet, we expected that an important motivation for them was that overweight negatively influenced the chances of becoming pregnant [3,4], but the struggle with infertility may have made lifestyle changes more difficult.

The most important strength of the current study was the data collection at four points in time within the frame of a RCT design using mixed models to analyze the data. By taking into account the within person dependency of the data, we were able to use all available data and not only data of the complete cases. Therefore, we have a study sample representing the whole study population instead of a selection.

The first limitation of our study is the use of a control group who promptly started with infertility treatment after randomization. This could influence our results in different directions. The patient information leaflet of the LIFEstyle study contained information on the adverse effects of overweight and obesity on women’s reproductive health, pregnancy, and pregnancy outcomes. This could explain the improvements in diet and physical activity in the control group. In addition, infertility treatment is associated with stress [40–42] and hormonal changes [43], which can influence diet and physical activity in different directions [44,45]. A second limitation is the use of self-reported questionnaires instead of objective measurements. Participation in the intervention could lead to social desirability bias, leading to over-reporting healthy behavior and underreporting unhealthy behavior [46–50]. If social desirability bias is present it is likely that it affected the results of the intervention group to a larger extent than of the control group, since women in the intervention group were actively motivated and educated on a healthier lifestyle. However, the intervention group lost significantly more weight compared to the control group [19]. It is therefore unlikely that the intervention effect on diet and physical activity is caused by social desirability bias alone. A third limitation is that the FFQ only asked about the food products the intervention was targeted on. Although we were able to evaluate whether the dietary intervention goals were achieved, we were not able to assess whether women replaced their sugary drinks and snacks with other (unhealthy) foods. Nor were we able to assess whether the intervention group lowered total energy intake compared to the control group or to correct for energy intake, since we have no data on caloric intake of the women randomized into the control group. It is however very likely that the intervention group did lower total energy intake since body weight decreased significantly compared to the control group.

In conclusion, we demonstrated that a six-month structured preconception lifestyle intervention in obese infertile women decreased the intake of unhealthy, high caloric foods and beverages and increased physical activity compared to the control group receiving prompt infertility treatment. These improvements in lifestyle, together with the improved cardiometabolic health, may in the future have beneficial effects on health of women and their offspring.

## Supporting information

S1 Table Sensitivity analyses of differences in fruit intake, sugary drinks, savory snacks, and sweet snacks in the intervention group compared to the control group. (DOCX)

S2 Table Dietary intake at baseline, three months, six months and twelve months after randomization. (DOCX)

S3 Table Moderate to vigorous physical activity (MVPA) at baseline, three months, six months and twelve months after randomization. (DOCX)

S1 File Trial protocol of the LIFEstyle study. (PDF)

S2 File CONSORT checklist. (PDF)

S3 File Minimal dataset. (SAV)
