# Supplementary material for: Effects of a preconception lifestyle intervention in obese infertile women on diet and physical activity; A secondary analysis of a randomized controlled trial
Source: PLoS One. 2018 Nov 7;13(11):e0206888. doi: 10.1371/journal.pone.0206888 (PMC6221548; doi:10.1371/journal.pone.0206888)
Supplement: S1 Table — (DOCX) [file pone.0206888.s001.docx]

**S1 Table.** Sensitivity analyses of differences in fruit intake, sugary drinks, savory snacks, and sweet snacks in the intervention group compared to the control group.

|  | **Overall  (95% C.I.)^a^** | **Time point after randomization** | **Difference  (95% C.I.)** | **P-value** |
| --- | --- | --- | --- | --- |
| **Fruit intake (gram/day)** | | | | |
| Corrected for baseline | -0.5  (-11.8; 10.8) | Three months | 7.2 (-6.8; 21.2) | 0.32 |
|  |  | Six months | -12.3 (-28.9; 4.2) | 0.14 |
|  |  | Twelve months | -0.7 (-19.6; 18.2) | 0.94 |
| Corrected for baseline, education, pregnancy and smoking | 0.7 (-10.8; 12.3) | Three months | 8.9 (-5.3; 23.1) | 0.22 |
|  |  | Six months | -8.7 (-25.5; 8.2) | 0.31 |
|  |  | Twelve months | -5.3 (-24.6; 14.0) | 0.59 |
| **Sugary drinks (glasses/day)** | | | | |
| Corrected for baseline | -0.4  (-0.7; -0.1)^c^ | Three months | -0.5 (-0.9; -0.2) | 0.003 |
|  |  | Six months | -0.4 (-0.9; -0.001) | 0.05 |
|  |  | Twelve months | 0.1 (-0.4; 0.6) | 0.70 |
| Corrected for baseline, education, pregnancy and smoking | -0.4 (-0.7; -0.1)^c^ | Three months | -0.5 (-0.9; -0.2) | 0.003 |
|  |  | Six months | -0.4 (-0.8; 0.1) | 0.11 |
|  |  | Twelve months | 0.03 (-0.5; 0.5) | 0.89 |
| **Savory snacks (handful/week)** | | | | |
| Corrected for baseline | -2.0  (-2.9; -1.1)^d^ | Three months | -2.7 (-3.8; -1.6) | <0.001 |
|  |  | Six months | -1.7 (-3.0; -0.4) | 0.01 |
|  |  | Twelve months | -0.7 (-2.3; 0.8) | 0.34 |
| Corrected for baseline, education, pregnancy and smoking | -2.0 (-2.9; -0.9)^d^ | Three months | -2.8 (-3.9; -1.6) | <0.001 |
|  |  | Six months | -1.5 (-2.9; -0.2) | 0.03 |
|  |  | Twelve months | -0.3 (-1.9; 1.2) | 0.68 |
| **Sweet snacks (portion/week)^b^** | | | | |
| Corrected for baseline | -2.0  (-3.0; -0.9)^d^ | Three months | -2.4 (-3.7; -1.2) | <0.001 |
|  |  | Six months | -1.3 (-2.8; 0.2) | 0.09 |
|  |  | Twelve months | -1.9 (-3.6; -0.2) | 0.03 |
| Corrected for baseline, education, pregnancy and smoking | -1.9 (-2.9; -0.8)^d^ | Three months | -2.4 (-3.6; -1.1) | <0.001 |
|  |  | Six months | -1.1 (-2.7; 0.4) | 0.16 |
|  |  | Twelve months | -1.9 (-3.7; -0.1) | 0.04 |

As all dietary questions contained open answer categories for the largest portion size, with the exception of vegetable intake, we performed a sensitivity analysis recoding this portion size into X+1+30% (see method section main article). Differences and 95% confidence intervals (95% CI) were analyzed by mixed model analysis, including all women with at least one value (range N=511 for sugary drinks; 535 for fruit intake), using a random intercept, including time and an interaction term between time and randomization group in all models. We corrected by default for baseline values, and in the fully corrected model for education, pregnancy and smoking; C.I.= confidence interval; min/week = minutes per week.

**^a^** The overall effect represents the effect of randomization group on the outcomes irrespective of the effect of time. The linear mixed model included randomization group, baseline dietary intake/physical activity, and in case of the fully corrected model, education level and pregnancy as independent fixed effect variables. Time was not added to this model.

^b^ One portion of sweet snacks included 2 biscuits, or 2 pieces of chocolate, or 5 candies, or 5 pieces of liquorice.

^c^ P-value <0.05
^d^ P-value <0.001
