# Supplementary material for: Effects of a preconception lifestyle intervention in obese infertile women on diet and physical activity; A secondary analysis of a randomized controlled trial
Source: PLoS One. 2018 Nov 7;13(11):e0206888. doi: 10.1371/journal.pone.0206888 (PMC6221548; doi:10.1371/journal.pone.0206888)
Supplement: S2 Table — (DOCX) [file pone.0206888.s002.docx]

**S2 Table.** Dietary intake at baseline, three months, six months and twelve months after randomization.

|  | **N** | **Intervention** | **N** | **Control** |
| --- | --- | --- | --- | --- |
| **Baseline** | | | | |
| Vegetable intake (g/day) | 258 | 114 (82; 157) | 248 | 125 (80; 171) |
| Fruit intake (g/day) | 258 | 86 (57; 171) | 246 | 86 (43; 143) |
| Sugary drinks (glasses/day) | 223 | 0.86 (0.22; 2.40) | 228 | 0.95 (0.25; 2.17) |
| Alcoholic beverages (glasses/day) | 237 | 0 (0; 0.18) | 238 | 0 (0; 0.18) |
| Savory snacks (handful/week) | 240 | 4.40 (1.76; 8.51) | 238 | 4.40 (1.76; 12.50) |
| Sweet snacks (portion/week)^a^ | 239 | 2.50 (0.88; 6.50) | 237 | 4.50 (0.88; 6.50) |
| **Three months after randomization** (median weeks [IQR]: 15 weeks [13-17]) | | | | |
| Vegetable intake (g/day) | 170 | 132 (93; 193) | 204 | 129 (88; 193) |
| Fruit intake (g/day) | 171 | 143 (71; 200) | 208 | 100 (57; 171) |
| Sugary drinks (glasses/day) | 146 | 0.43 (0.14; 1.15) | 178 | 0.86 (0.29; 1.97) |
| Alcoholic beverages (glasses/day) | 159 | 0 (0; 0.08) | 189 | 0 (0; 0.08) |
| Savory snacks (handful/week) | 160 | 1.76 (0; 4.40) | 187 | 1.90 (1.76; 5.0) |
| Sweet snacks (portion/week)^a^ | 160 | 0.88 (0.38; 2.50) | 188 | 2.50 (0.88; 6.50) |
| **Six months after randomization** (median weeks [IQR]: 28 weeks [26-31]) | | | | |
| Vegetable intake (g/day) | 104 | 129 (86; 171) | 146 | 129 (86; 171) |
| Fruit intake (g/day) | 111 | 143 (57; 171) | 147 | 143 (57; 200) |
| Sugary drinks (glasses/day) | 88 | 0.54 (0.14; 1.13) | 128 | 0.93 (0.29; 1.82) |
| Alcoholic beverages (glasses/day) | 100 | 0 (0; 0.08) | 139 | 0 (0; 0) |
| Savory snacks (handful/week) | 100 | 1.76 (0.76; 4.40) | 139 | 1.76 (0.76; 5.0) |
| Sweet snacks (portion/week)^a^ | 99 | 1.76 (0.88; 2.50) | 136 | 2.50 (0.88; 5.00) |
| **Twelve months after randomization** (median weeks [IQR]: 57 weeks [53; 60] | | | | |
| Vegetable intake (g/day) | 77 | 129 (86; 182) | 107 | 129 (86; 171) |
| Fruit intake (g/day) | 80 | 143 (71; 200) | 105 | 114 (57; 186) |
| Sugary drinks (glasses/day) | 65 | 0.61 (0.14; 1.66) | 95 | 0.86 (0.29; 2.00) |
| Alcoholic beverages (glasses/day) | 71 | 0 (0; 0) | 97 | 0 (0; 0) |
| Savory snacks (handful/week) | 70 | 1.90 (0.76; 4.55) | 99 | 3.04 (0.76; 7.04) |
| Sweet snacks (portion/week)^a^ | 72 | 2.50 (0.88; 5.00) | 95 | 2.50 (1.76; 6.50) |

All values are medians (inter quartile ranges); g/day = gram per day; IQR = inter quartile ranges.

^a^ One portion of sweet snacks included 2 biscuits, or 2 pieces of chocolate, or 5 candies, or 5 pieces of liquorice.
