# Supplementary material for: Effects of a preconception lifestyle intervention in obese infertile women on diet and physical activity; A secondary analysis of a randomized controlled trial
Source: PLoS One. 2018 Nov 7;13(11):e0206888. doi: 10.1371/journal.pone.0206888 (PMC6221548; doi:10.1371/journal.pone.0206888)
Supplement: S3 Table — (DOCX) [file pone.0206888.s003.docx]

**S3 Table.** Moderate to vigorous physical activity (MVPA) at baseline, three months, six months and twelve months after randomization.

|  | **N** | **Intervention** | **N** | **Control** |
| --- | --- | --- | --- | --- |
| **Baseline** | | | | |
| Total MVPA (min/week) | 255 | 350 (125; 900) | 243 | 404 (150; 1140) |
| Leisure time MVPA (min/week) | 255 | 180 (20; 340) | 243 | 160 (60; 360) |
| Commuting MVPA (min/week) | 255 | 0 (0; 40) | 243 | 0 (0; 90) |
| **Three months after randomization** (median weeks [IQR]: 15 weeks [13-17]) | | | | |
| Total MVPA (min/week) | 166 | 473 (240; 1215) | 204 | 420 (120; 960) |
| Leisure time MVPA (min/week) | 166 | 240 (120; 469) | 204 | 180 (41; 360) |
| Commuting MVPA (min/week) | 166 | 0 (0; 33) | 204 | 0 (0; 60) |
| **Six months after randomization** (median weeks [IQR]: 28 weeks [26-31]) | | | | |
| Total MVPA (min/week) | 108 | 360 (153; 775) | 146 | 260 (60; 865) |
| Leisure time MVPA (min/week) | 108 | 203 (60; 368) | 146 | 120 (0; 270) |
| Commuting MVPA (min/week) | 108 | 0 (0; 55) | 146 | 0 (0; 46.3) |
| **Twelve months after randomization** (median weeks [IQR]: 57 weeks [53.5; 60.5]) | | | | |
| Total MVPA (min/week) | 78 | 180 (56; 664) | 107 | 150 (0; 600) |
| Leisure time MVPA (min/week) | 78 | 60 (0; 251) | 107 | 60 (0; 240) |
| Commuting MVPA (min/week) | 78 | 0 (0; 20) | 107 | 0 (0; 0) |

All values are medians (inter quartile ranges), otherwise specified; min/week = minutes per week; IQR = inter quartile ranges.
